# Novel Hybrid Brain-Computer Interface for Virtual Reality Applications Using Steady-State Visual-Evoked Potential-Based Brain–Computer Interface and Electrooculogram-Based Eye Tracking for Increased Information Transfer Rate

**DOI:** 10.3389/fninf.2022.758537

**Published:** 2022-02-24

**Authors:** Jisoo Ha, Seonghun Park, Chang-Hwan Im

**Affiliations:** ^1^Department of HY-KIST Bio-Convergence, Hanyang University, Seoul, South Korea; ^2^Department of Electronic Engineering, Hanyang University, Seoul, South Korea; ^3^Department of Biomedical Engineering, Hanyang University, Seoul, South Korea

**Keywords:** brain-computer interface, electroencephalogram, electrooculogram, virtual reality, steady state visual evoked potential

## Abstract

Brain–computer interfaces (BCIs) based on electroencephalogram (EEG) have recently attracted increasing attention in virtual reality (VR) applications as a promising tool for controlling virtual objects or generating commands in a “hands-free” manner. Video-oculography (VOG) has been frequently used as a tool to improve BCI performance by identifying the gaze location on the screen, however, current VOG devices are generally too expensive to be embedded in practical low-cost VR head-mounted display (HMD) systems. In this study, we proposed a novel calibration-free hybrid BCI system combining steady-state visual-evoked potential (SSVEP)-based BCI and electrooculogram (EOG)-based eye tracking to increase the information transfer rate (ITR) of a nine-target SSVEP-based BCI in VR environment. Experiments were repeated on three different frequency configurations of pattern-reversal checkerboard stimuli arranged in a 3 × 3 matrix. When a user was staring at one of the nine visual stimuli, the column containing the target stimulus was first identified based on the user’s horizontal eye movement direction (left, middle, or right) classified using horizontal EOG recorded from a pair of electrodes that can be readily incorporated with any existing VR-HMD systems. Note that the EOG can be recorded using the same amplifier for recording SSVEP, unlike the VOG system. Then, the target visual stimulus was identified among the three visual stimuli vertically arranged in the selected column using the extension of multivariate synchronization index (EMSI) algorithm, one of the widely used SSVEP detection algorithms. In our experiments with 20 participants wearing a commercial VR-HMD system, it was shown that both the accuracy and ITR of the proposed hybrid BCI were significantly increased compared to those of the traditional SSVEP-based BCI in VR environment.

## Introduction

Brain–computer interface (BCI) is a technology that directly translates brain activities into commands to provide users with a new communication channel toward outside world (Vallabhaneni et al., 2013). Although previous BCIs were mostly applied to patients with severe paralyzes (Daly and Wolpaw, 2008), their applications have been gradually expanded to the general public in recent years, one of the representative example of which includes hands-free operation of external devices (Coogan and He, 2018) such as drones (Nourmohammadi et al., 2018), robots (Liu et al., 2021), and game characters (Vasiljevic and De Miranda, 2019). Recently, the rapid development of virtual reality (VR) technology brought about the increasing demand for new types of input devices for VR applications, other than the conventional hand controllers, particularly for those who cannot freely move their limbs due to severe paralyzes. For the hands-free control of VR or augmented reality (AR) devices, electroencephalography (EEG)-based BCIs have been intensively studied because of its affordability, ease of use, and portability (Park et al., 2019). Among the various EEG-based BCI paradigms including motor imagery (MI), P300, and steady-state visual-evoked potential (SSVEP) (Yin et al., 2015), SSVEP-based BCIs have been successfully applied to VR applications as a new hands-free communication tool, thanks to its high information transfer rate (ITR) and no (or less) training requirement (Coogan and He, 2018; Stawicki et al., 2018; Choi et al., 2019; Grichnik et al., 2019; Monteiro et al., 2021). However, the number of visual stimuli used for SSVEP-based BCIs in VR environment was generally limited to four or five because of the relatively poorer performance of the SSVEP-based BCIs implemented using head-mounted displays (HMDs) than those implemented with conventional liquid crystal displays (LCDs) (Chen et al., 2015; Yao et al., 2018). As will be shown in the results of this study, the increment of the number of visual stimuli (e.g., 9) greatly degrades the overall classification accuracy compared to previous studies because the SSVEP-based BCIs implemented with VR-HMDs are more prone to be affected by the peripheral vision due to the relatively short distance between the eyes and the display.

To effectively increase the number of target visual stimuli and/or elevate the overall performance of BCIs, a variety of hybrid BCIs combining EEG with other physiological signals, including video-oculography (VOG) (Hong and Khan, 2017; Stawicki et al., 2017; Ma et al., 2018; Yao et al., 2018) and electrooculogram (EOG) (Saravanakumar and Ramasubba Reddy, 2019, 2020), have been developed. VOG is a non-invasive, video-based method to measure the movements of the eyes and is known to provide a high-precision estimate of the absolute coordinates of the eye gaze. However, because the amount of light inside the VR-HMDs is often too limited to track the pupil of the eyes, most VOG-based eye trackers embedded into VR-HMDs are implemented with infrared wide-angle cameras (Clay et al., 2019; Sipatchin et al., 2021), which leads to a rise in the total device cost (Ivanchenko et al., 2021; Modi and Singh, 2021). On the contrary, the eye trackers based on EOG can be implemented at a much lower cost than VOG-based eye trackers (Chang, 2019). Moreover, the EOG signals can be recorded using the same amplifier for recording EEG, whereas the VOG systems are totally independent from the EEG systems, which increases the overall expense of the system. Specifically, VIVE Pro Eye, a commercialized VR-HMD combined with a camera-based eye tracker, costs about 1,500 USD.^1^ Because the VR-HMD costs approximately 700 USD per device, it can be said that adding a VOG device costs approximately 800 USD. On the contrary, as the EOG shares the same amplifier with EEG, just adding two EOG electrodes would cost less than 100 USD, far lower price than VOG.

However, in most hybrid BCIs that simultaneously used EOG and EEG, EOG was employed just as an auxiliary tool to switch on/off SSVEP-based BCIs by identifying the eye blinks of the users (Park et al., 2019; Saravanakumar and Ramasubba Reddy, 2019; Zhou et al., 2020; Zhu et al., 2020). There was only one previous study that used EOG-based eye tracking to select a group of targets in an SSVEP-based BCI (Saravanakumar and Ramasubba Reddy, 2020) although the study used an LCD for the rendering device. Their system consisted of two sequential steps: (i) eye movements or blinks detected from EOG were used to select one of the nine target groups, and then, (ii) SSVEP was used for detecting a target among four visual stimuli included in the group. Because the system did not simultaneously use EOG with EEG, it is not only questionable whether their system can be classified as a hybrid BCI system, but also the time required for a single selection process was relatively long compared to the other hybrid BCI systems owing to the two-step selection process.

In this study, we proposed a novel hybrid BCI system that simultaneously use EOG-based eye tracking with SSVEP-based BCIs with the aim to improve the BCI performance of a nine-target SSVEP-based BCI in VR-HMD environment. Inspired by the fact that horizontal EOG (hEOG) recorded from a pair of electrodes attached to outer canthi of both eyes can be classified with a high classification accuracy, one of the three columns in a 3 × 3 matrix was first identified based on the user’s horizontal eye movement direction (left, middle, or right). Note that vertical EOG (vEOG) was not employed in our study because the estimation of eye gaze using vEOG is generally known to be less accurate than that using hEOG (Chang et al., 2016; Lee et al., 2017). Then, the target visual stimulus was identified among the three pattern-reversal checkerboard stimuli vertically arranged in the selected column based on the SSVEP-based BCI. Unlike the previous study by Saravanakumar and Ramasubba Reddy (2020), we used the EOG and EEG signals simultaneously to select a single target among nine visual stimuli. Through experiments with 20 participants wearing a commercial VR-HMD system, it was investigated whether the BCI performance of the proposed hybrid BCI could be significantly enhanced compared to that of the conventional SSVEP-based BCI in VR environment.

## Materials and Methods

### Participants

A total of 20 healthy volunteers (16 men and 4 women, 25 ± 3.4 years) with normal or corrected-to-normal vision participated in the experiment. None of the participants reported a history of neurological or psychiatric disorders that might have influenced the experimental results. However, the data of two participants were excluded owing to the nonexistence of spectral peaks in the recorded EEG; thereby, data of 18 participants were analyzed in the further analyses. This so-called BCI-illiteracy is a well-reported issue in the EEG-based BCIs (Allison et al., 2010). All participants were given monetary compensation for their participation in the experiments. The study protocol was approved by the Institutional Review Board (IRB) of Hanyang University, South Korea (IRB no. HYI-14-167-13).

### Apparatus

**FIGURE 1:**
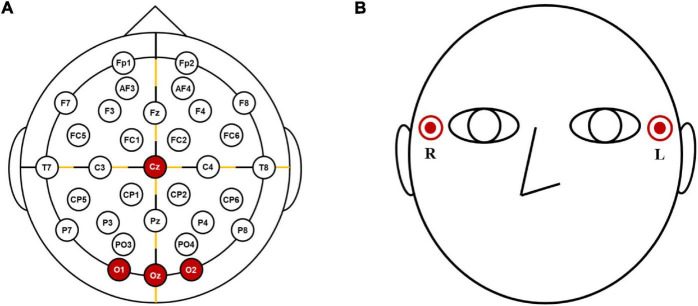
The configurations of the electrodes: **(A)** locations of EEG electrodes and **(B)** EOG electrodes.

The EEG and EOG data were recorded from four electrodes attached to the scalp (Cz, O1, Oz, and O2) (Figure 1A) and two electrodes attached to the outer canthi of both eyes (see Figure 1B), respectively, using a multichannel biosignal recording system (BioSemi ActiveTwo, Amsterdam, the Netherlands) at a sampling rate of 2,048 Hz. A common mode sense electrode and a driven right leg electrode were placed at left and right mastoids, respectively. Participants were asked to refrain from any voluntary movement during the entire experiment.

Visual stimuli were presented in a VR environment using a commercial VR-HMD (VIVE*™*; HTC Co. Ltd., NewTaipei City, Taiwan), whose refresh rate was set to 90 Hz. To keep the constancy of the relative position between the participant and the visual stimuli, the screen on the VR-HMD was set to follow the head movement of the participant. In other words, the screen presented in the front of the participant in VR environment remained the same regardless of the user’s head movement. The wearing angle of the VR-HMD and its interpupillary distance (IPD) was adjusted for each participant before the main experiment.

### Experiment Paradigm

**FIGURE 2:**
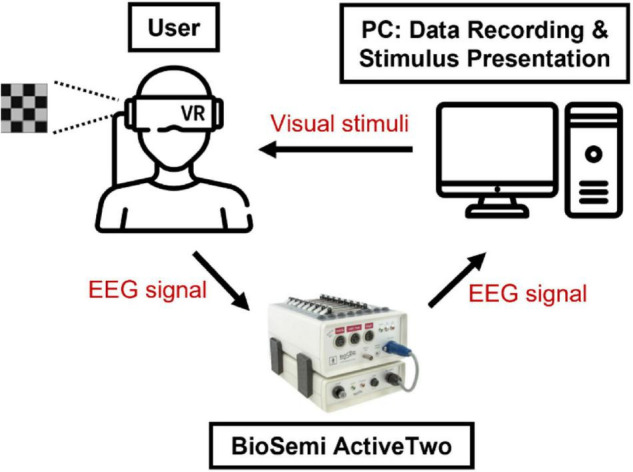
A schematic diagram of the proposed hybrid BCI system. The visual stimuli were presented to the user *via* a VR-HMD, which was controlled by Unity software in a PC. The EEG signals were measured from a user by a biosignal amplifier (BioSemi ActiveTwo).

A schematic diagram of the proposed hybrid BCI system is presented in Figure 2. The visual stimuli were presented to the user via a VR-HMD, which was controlled by Unity software in a PC. The EEG signals were measured from the user by a biosignal amplifier (BioSemi ActiveTwo).

**FIGURE 3:**
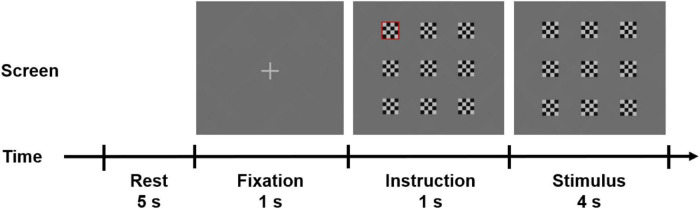
Schematic diagram of a single trial in the experimental paradigm.

Each trial consisted of rest (5 s), fixation (1 s), instruction (1 s), and stimulus (4 s) periods (see Figure 3). After the rest period, the participants were instructed to stare at the fixation cross on the center of the HMD screen (denoted by the fixation period), followed by the instruction period when one visual stimulus among the nine stimuli was highlighted with a red rectangular boundary to let the participant know the location of the target visual stimulus to stare at during the stimulus period. The participants were asked to restrain from eye blinking during the fixation, instruction, and stimulus periods, but they could blink their eyes during the rest period. The experiment paradigm was developed using Unity 3D (Unity Technologies ApS, San Francisco, CA, United States) and was presented on the VR-HMD.

**FIGURE 4:**
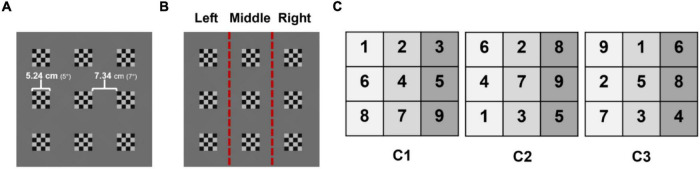
**(A)** Nine visual stimuli presented on the screen, **(B)** the three directions classified using EOG: left, middle, and right, **(C)** three different configurations of pattern-reversing frequencies. Numbers represent the stimulus numbers listed in Table 1. C1, C2, and C3 represent the three configurations of visual stimuli tested in the experiments.

In the stimulus period, a 3 × 3 array of pattern-reversal checkerboard (PRC) stimuli was used to elicit SSVEP responses. The visual stimuli presented on the VR-HMD are shown in Figure 4A. Total nine checkerboard stimuli reversing at different frequencies were presented, whose reversing frequencies are listed in Table 1. The frequencies were determined by dividing the refreshing rate of VR-HMD (90 Hz) by natural numbers ranging from 7 to 15. The distance between the eye and the stimuli was set to 60 cm in the virtual environment. Therefore, the visual angle of each stimulus was set to 5° (5.24 cm), and the visual angle between two adjacent stimuli was set to 7° (7.34 cm). Each of the nine visual stimuli was repeatedly selected three times in a randomized order for each participant; therefore, total 27 trials were performed for each session. Three different configurations of the nine visual stimuli were tested to rule out the variation of classification accuracy according to the stimuli arrangements (see Figure 4C, where the three configurations are denoted by C1, C2, and C3 and the number in each element of the matrix indicates the stimulus number in Table 1). Each stimulus configuration was made according to a rule that stimuli with similar frequencies or overlapping harmonic components should not be placed closely with each other. To rule out the potential influence of the experimental order, the three different configurations were presented in a randomized order for each participant.

**TABLE 1:** Pattern-reversing frequencies used for the experiments.

Stimulus number	1	2	3	4	5	6	7	8	9
Freq (Hz)	12.86	11.25	10.00	9.00	8.18	7.50	6.92	6.43	6.00

*The stimulus numbers (1–9) correspond to the numbers in the stimulus configurations depicted in Figure 4C.*

### Preprocessing of Electroencephalogram and Electrooculogram Signals

The recorded EEG and EOG data were downsampled from 2,048 to 512 Hz to reduce the computational cost. The EEG data were bandpass filtered with 2 and 54 Hz cutoff frequencies, using a sixth order zero-phase Butterworth filter, to remove power-line noise (60 Hz) and low-frequency fluctuations. The filtered EEG data were then rereferenced with respect to Cz, by subtracting the EEG data of Cz from the EEG data of O1, Oz, and O2, respectively (Lai et al., 2011; Park et al., 2019). As for the EOG data, hEOG signal was extracted by subtracting the EOG signal of the left electrode from that of the right electrode. Then, the hEOG was lowpass filtered with a 10 Hz cutoff frequency, using a sixth-order zero-phase Butterworth filter. MATLAB R2020b (MathWorks; Natick, MA, United States) was used for the preprocessing of the recorded EEG and EOG data.

### Data Analysis

#### Electrooculogram Analysis

Horizontal EOG was used to classify three directions of the eye movement: left, middle, and right (Figure 4B). A simple algorithm was developed to classify the eye movement directions with a high classification accuracy without a need for individual calibration sessions. First of all, baseline correction was performed by subtracting the median of the hEOG recorded during the fixation period from the hEOG data recorded during the stimulus period. The median value was used owing to its superior performance of preserving edge steepness of saccadic eye movements (Bulling et al., 2011). The median of hEOG during the first 1 s of the stimulus period (hereafter denoted by *M*) was then evaluated for each trial. If the *M*-value dropped below −65 μV, the eye movement during the stimulus period was classified as “left,” whereas the eye movement was classified as “right” if the *M*-value exceeded 75 μV. Otherwise, the eye movement was classified as “middle.” The positive and negative threshold values (-65 and 75 μV, respectively) were determined using preliminary experiments with three participants and were applied to all the participants without any individual calibration process. Please note that these threshold values should be differently set if the experimental environments (e.g., recording device and visual angles between stimuli) are changed.

#### Electroencephalogram Analysis

After the classification of three directions, SSVEP responses were classified to one of the three PRC stimuli in the selected direction using the extension of multivariate synchronization index (EMSI) with no training (Zhang et al., 2017). Multivariate synchronization index (MSI) is an algorithm that finds the specific stimulation frequency that maximizes the synchronization index, by calculating the synchronization between a given EEG signal and reference signals generated with the stimulation frequencies and their harmonics. EMSI is an extended version of MSI to improve the performance of MSI by incorporating the first-order time-delayed version of the EEG data. In this study, two harmonic frequencies were taken into account, and none of the subharmonic components was considered. For the classification of SSVEP responses, three channels (O1, Oz, and O2) located in the occipital area were used.

### Performance Evaluation

The performance of the proposed hybrid BCI system was evaluated by comparing the BCI system simultaneously using SSVEP and EOG (hereafter denoted by the “SSVEP+EOG” case) with the BCI system using SSVEP responses only (hereafter denoted by the “SSVEP-only” case). To compare the performances for SSVEP+EOG with SSVEP-only cases, classification accuracy and ITR were evaluated for all three configurations (C1, C2, and C3) with respect to different analysis window sizes. The formula to calculate the ITR is given as follows:


(1) $$\text{ITR}=\frac{60}{T}\left({\text{log}}_{2}N+P{\text{log}}_{2}P\left(1-P\right){\text{log}}_{2}\left(\frac{1-P}{N-1}\right)\right),$$

where *T* represents the window size, *N* represents the number of possible targets, and *P* denotes the classification accuracy (Wolpaw et al., 1998). In addition, confusion matrices were computed to investigate the origin of the misclassification. To compare the difference in classification accuracies of SSVEP+EOG and SSVEP-only cases for different window sizes, statistical analyses were conducted using the Bonferroni-corrected Wilcoxon signed-rank test. Wilcoxon signed-rank test was employed as the classification accuracy, and ITR did not follow a normal distribution for both SSVEP-only and SSVEP+EOG cases.

## Results

**FIGURE 5:**
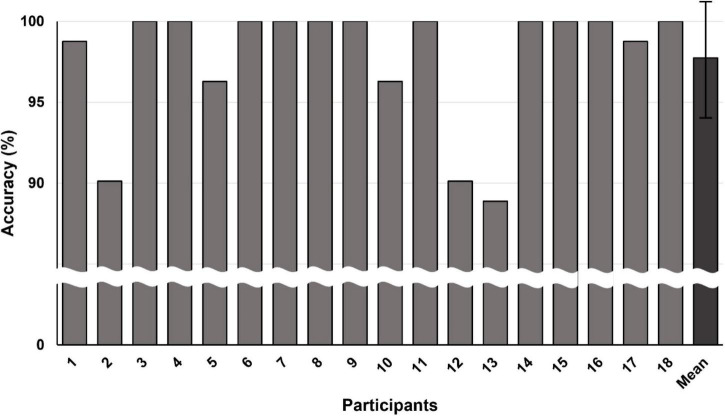
Accuracy of classifying three directions (left, middle, and right) using hEOG for each participant. The grand averaged classification accuracy was 97.74 ± 3.89% (denoted by “Mean” in the graph).

We first investigated whether the accuracy of classifying three directions (left, middle, and right) using hEOG was high enough to be employed for our hybrid BCI system (see Figure 5). The average classification accuracy was reported to be 97.74 ± 3.89%. Particularly, the classification accuracies for 11 out of 18 participants were 100%. These results suggest that the reliability of the hEOG-based eye tracking is high enough to be employed as a reliable tool for the recognition of horizontal eye movement directions.

**FIGURE 6:**
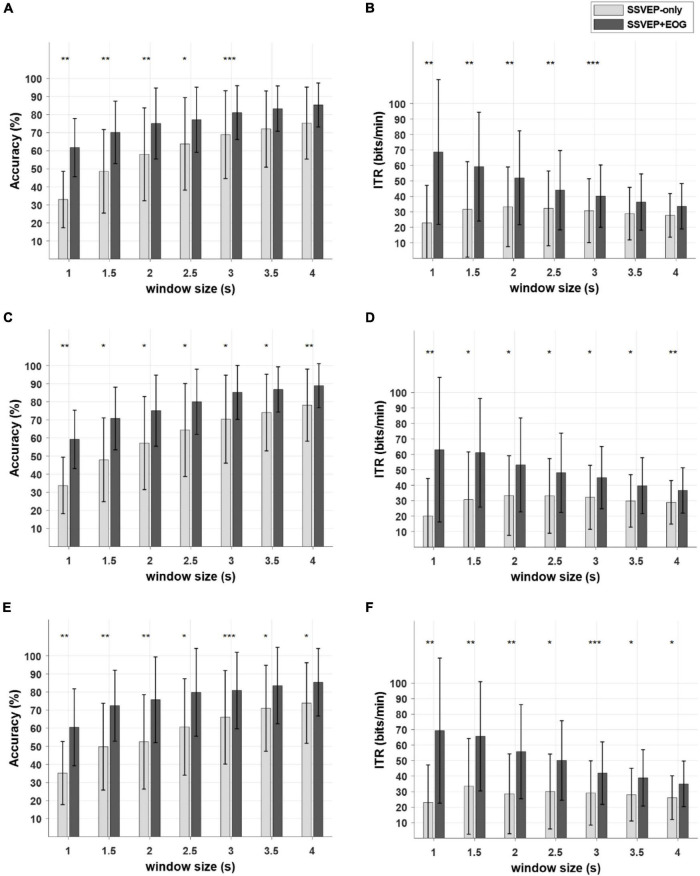
Comparison of the mean classification accuracies and information transfer rates (ITRs) for SSVEP-only and SSVEP+EOG cases with respect to different window sizes. Light gray and dark gray bars indicate SSVEP-only and SSVEP+EOG cases, respectively. Error bars indicate the standard deviation across the participants. **(A)** the accuracy of the C1, **(B)** the ITR of C1, **(C)** the accuracy of C2, **(D)** the ITR of C2, **(E)** the accuracy of C3, **(F)** the ITR of C3. (*p < 0.05, **p < 0.01, ***p < 0.005, Wilcoxon signed rank test, Bonferroni-corrected).

The comparison among all three frequency configurations (i.e., C1, C2, and C3 in Figure 4C) is presented in Supplementary Figure 1. As seen in the figures, no distinct trend was found across the results for three different frequency configurations. The same results were visualized in a different manner in Figure 6, where the mean classification accuracies and averaged ITR values were depicted as a function of analysis window sizes for SSVEP-only and SSVEP+EOG cases for each of three configurations. For all three configurations, the proposed hybrid BCI outperformed the conventional SSVEP-based BCI in terms of both the mean classification accuracy and ITR.

Statistical analyses also confirmed the superiority of the proposed hybrid BCI to the conventional SSVEP-based BCI. The SSVEP+EOG case outperformed the SSVEP-only case in the classification accuracy in (i) C1 for all window sizes except 3.5 and 4 s (Figure 6A: 1 s: *p* < 0.01; 1.5 s: *p* < 0.01; 2 s: *p* < 0.01; 2.5 s: *p* < 0.05; 3 s: *p* < 0.005; 3.5 s: *p* = 0.164; 4 s: *p* = 0.123), (ii) C2 for all window sizes (Figure 6C: 1 s: *p* < 0.01; 1.5 s: *p* < 0.05; 2 s: *p* < 0.05; 2.5 s: *p* < 0.05; 3 s: *p* < 0.05; 3.5 s: *p* < 0.05; 4 s: *p* < 0.01), and (iii) C3 for all window sizes (Figure 6E: 1 s: *p* < 0.01; 1.5 s: *p* < 0.01; 2 s: *p* < 0.01; 2.5 s: *p* < 0.05; 3 s: *p* < 0.005; 3.5 s: *p* < 0.05; 4 s: *p* < 0.05). As for the ITR, statistical significance was found in all three configurations except for the 3.5 and 4 s for window sizes in the C1 configuration (Figures 6B,D,F).

## Discussion

In this study, we proposed a new hybrid BCI system combining SSVEP-based BCI and EOG-based eye tracking to effectively improve the BCI performance in VR environment. Since only a single amplifier system is sufficient to record both EEG and EOG signals, the proposed hybrid BCI system can be made more concisely and economically compared to the conventional hybrid BCI systems combining EEG and VOG. Experiments were performed with three different frequency configurations of pattern-reversal checkerboard stimuli arranged in a 3 × 3 matrix displayed using a commercial VR-HMD. First, the column containing the target stimulus was identified based on the user’s horizontal eye movement direction (left, middle, or right) identified using horizontal EOG. The target visual stimulus was then identified among the three stimuli vertically arranged in the selected column using the EMSI algorithm. Our experimental results demonstrated that the proposed hybrid BCI could exhibit significantly higher classification accuracy and ITR than the conventional BCI that used SSVEP only. It is noteworthy that the proposed system does not require any calibration session or a trained classifier. To the best of our knowledge, this is the first study that implemented a hybrid BCI system by combining SSVEP and EOG without any additional tasks or training sessions. The results of experiments with 18 participants suggest that the proposed system has the potential to be used as a practical hands-free control tool for VR applications, particularly for those who cannot freely move their limbs.

To further investigate the origin of errors in the BCI systems tested in our experiments, confusion matrices were evaluated for the SSVEP-only case (Supplementary Figure 2) and also the SSVEP+EOG case (Supplementary Figure 3). As shown in the confusion matrix for the SSVEP-only case, the misclassification occurred most frequently between the frequency of 12.86 Hz and 6.43 Hz, which can be observed in (1, 7), (7, 3), and (2, 6) elements of C1, C2, and C3 confusion matrices, respectively, in the Supplementary Figure 2. These misclassifications occurred because these two frequencies share some harmonic frequency components. However, when these two frequencies were placed in different columns (C2 and C3 configurations), the employment of the proposed hybrid BCI method could dramatically prevent the misclassification between these two frequencies (13.2% → 0% in C2-2s case; 20.0% → 0% in C2-4s case; 26.5% → 0% in C3-2s case; and 26.7% → 0% in C3-4s case) (see Supplementary Figure 3). It is interesting to note that the misclassification rate for these two frequencies was still high in the case of C1 configuration where both frequencies were placed in the same column. These results suggest that an appropriate arrangement of the stimulation frequencies would help to increase the overall classification accuracy of the hybrid BCI system. Indeed, as seen from Supplementary Figure 1, the configuration C1 did not show the best performances among the three configurations for any window sizes in the SSVEP+EOG case. In contrast, C2 showed the best performances among the three, by avoiding the frequencies that have overlapping harmonic components.

In this study, only hEOG was used to classify the horizontal eye movement directions: left, middle, and right. vEOG was not employed in this study owing to its instability. vEOG is known to be easily biased by sweat-oriented noises in the forehead or facial asymmetry (Chang et al., 2016), which was the main reason why we excluded vEOG in this study. As demonstrated in our experimental results, the hEOG could be used to determine the eye movement directions with a high accuracy without an individual calibration session. This aspect is meaningful in that VOG-based eye trackers commonly employ 5-point calibration as a standard, which takes approximately 19 s (Saha et al., 2021). It is obviously a cumbersome task for the users to conduct the calibration every time they use the device.

However, there still remains an issue to be addressed in implementing the practical EOG-based eye-tracking systems: To obtain a stable hEOG signal to precisely estimate the horizontal eye movement directions, an hEOG signal recorded for a short period of time before the main execution period might be necessary for the baseline correction process. This implies that the users of the hybrid BCI system should always keep their eye gaze to the center of the screen for a short period of time before every trial onset. This baseline correction process is necessary because there can be a low-frequency fluctuation in the EOG signal, particularly by the sweat noise (Lee et al., 2017), which depends on the recording environment. Nevertheless, starting eye gazing from the center of the screen itself is not a task to increase the user’s fatigue; therefore, it does not seem to be a critical problem. In our future study, we would consider an extended version of real-time hybrid BCIs by measuring vEOG channels to detect eye blinks that would be used to call the initial screen. This process would not only work as an on/off switch of the SSVEP-based BCIs, but also would eliminate the baseline correction process.

In this study, we used a commercial high-end EEG recording device to record both EEG and EOG signals, which could provide high-quality signals but generally too expensive to be used for general consumers. With the recent advancement of portable EEG technology (Casson, 2019), dry EEG and/or EOG electrodes are integrated with VR-HMD (Cattan et al., 2020). Indeed, some commercial VR-HMD systems with EEG and/or EOG recording modules are already available in market (e.g., Looxid Labs Inc.).^2^ Additionally, a recently developed wearable EEG device named NextMind™,^3^ which can record SSVEP responses with dry electrodes attached to the occipital area, also showed a potential to be readily incorporated with existing VR-HMDs. We expect that our proposed hybrid BCI system can be implemented with a wearable biosignal recording system incorporated with commercial VR-HMD systems, in the near future. In addition, although we rereferenced EEG data with respect to Cz, other studies applied different bipolar settings to get an improved performance of the SSVEP-based BCIs (Diez et al., 2010; Müller et al., 2015). In our future study, we will try to increase the overall performance of the proposed system by employing various bipolar settings.

With the recent development of emerging metaverse technologies, the application fields of VR have been expanding rapidly. Nowadays, BCI technologies are actively studied as a new modality of communication in various VR applications (Coogan and He, 2018), including rehabilitation (Karamians et al., 2020), medical training (Singh et al., 2020), entertainment (Liu et al., 2020), and education (Sundaram et al., 2020). Our proposed hybrid BCI technology that outperformed the conventional SSVEP-based BCI in terms of classification accuracy and ITR is expected to be used as an important tool for hands-free controlling of VR environments especially for those who cannot freely move their limbs. Furthermore, this work also suggests its potential impact on neuro-rehabilitation by allowing at-home use.

## Data Availability Statement

The raw data supporting the conclusions of this article will be made available by the authors, without undue reservation.

## Ethics Statement

The studies involving human participants were reviewed and approved by the Institutional Review Board (IRB) of Hanyang University. The patients/participants provided their written informed consent to participate in this study.

## Author Contributions

JH designed and performed experiments, developed the algorithm, and analyzed the data. SP suggested the idea and gave the advice. C-HI supervised the study. JH and C-HI wrote the manuscript. All authors reviewed the manuscript.

## Conflict of Interest

The authors declare that the research was conducted in the absence of any commercial or financial relationships that could be construed as a potential conflict of interest.

## Publisher’s Note

All claims expressed in this article are solely those of the authors and do not necessarily represent those of their affiliated organizations, or those of the publisher, the editors and the reviewers. Any product that may be evaluated in this article, or claim that may be made by its manufacturer, is not guaranteed or endorsed by the publisher.

## Footnotes

1
https://www.vive.com/uk/product/vive-pro-eye/overview/

2
https://looxidlabs.com/looxidlink

3
https://www.next-mind.com
