# Supplementary material for: Novel Hybrid Brain-Computer Interface for Virtual Reality Applications Using Steady-State Visual-Evoked Potential-Based Brain–Computer Interface and Electrooculogram-Based Eye Tracking for Increased Information Transfer Rate
Source: Front Neuroinform. 2022 Feb 24;16:758537. doi: 10.3389/fninf.2022.758537 (PMC8908008; doi:10.3389/fninf.2022.758537)

Supplementary Material

Supplementary Figure 1.

The classification accuracies and ITRs obtained using only SSVEP (SSVEP-only) and using both SSVEP and EOG (SSVEP+EOG) for three configurations (C1, C2, and C3 in Figure 3C), with respect to different analysis window sizes.

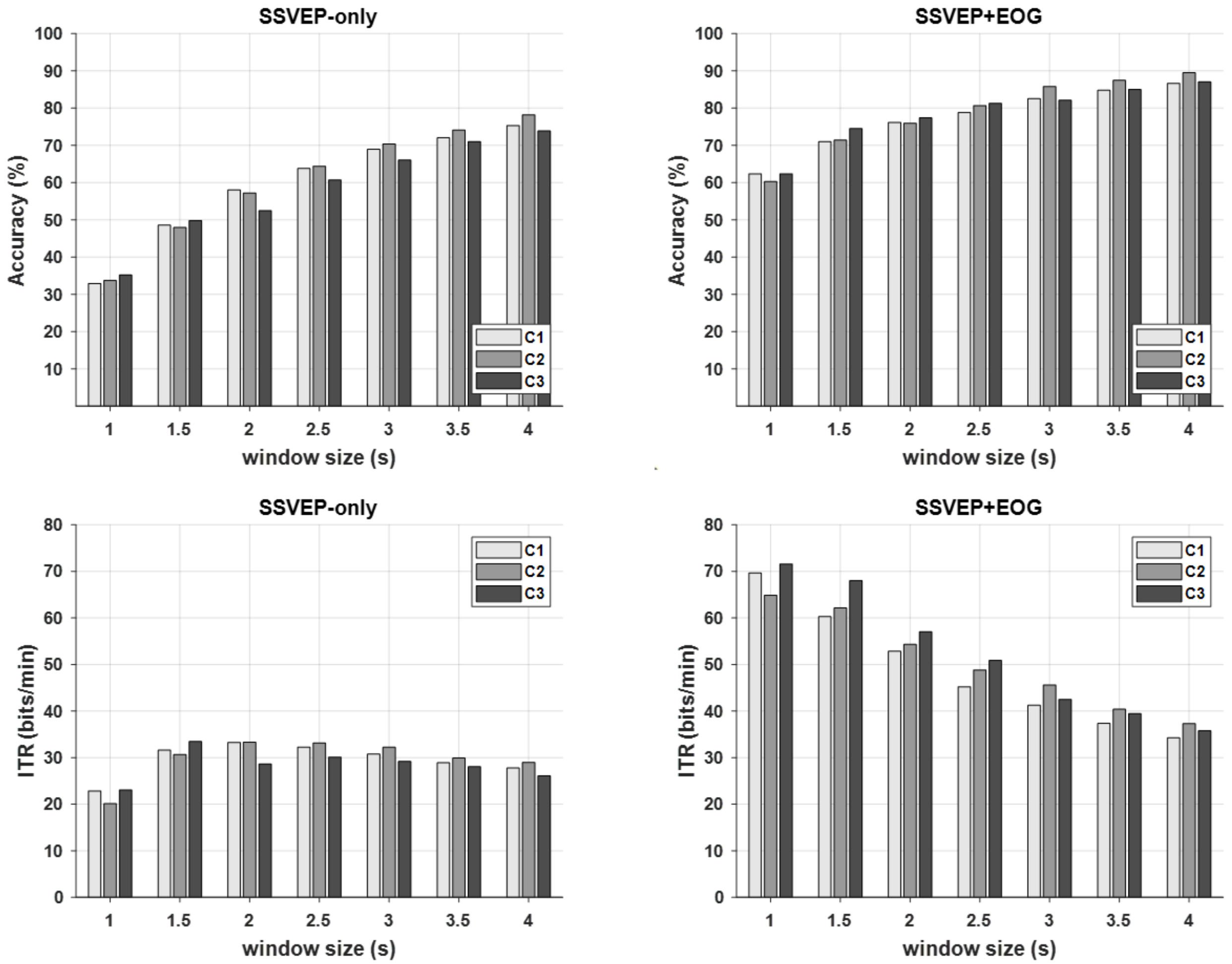

Supplementary Figure 2.

Confusion matrices of the SSVEP-only case. The class numbers (output class and target class) in the horizontal and vertical axes indicate the position illustrated at the top panel of the figures. C1, C2, and C3 represent the frequency configurations depicted in Figure 3C.

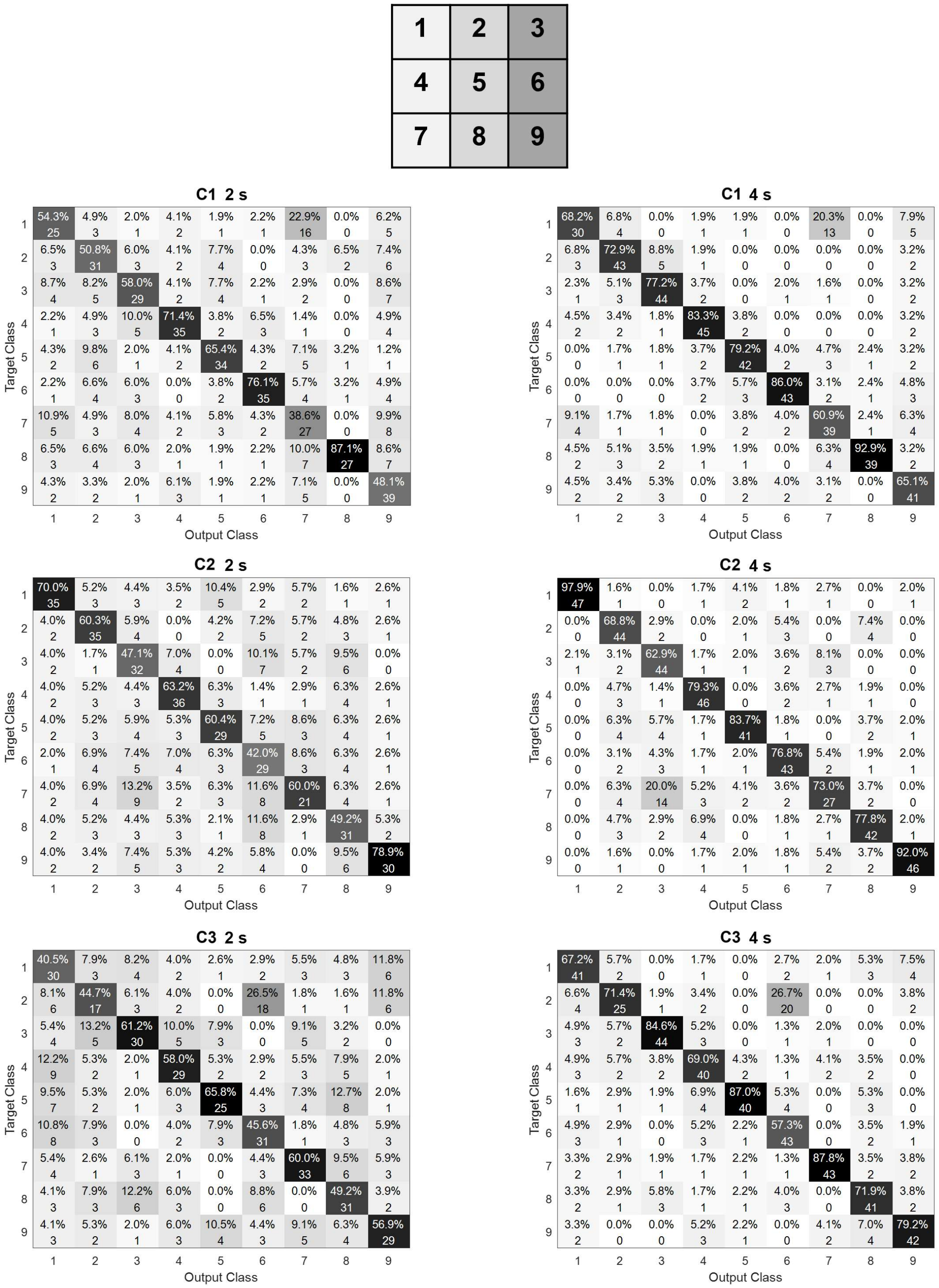

Supplementary Figure 3.

Confusion matrices of the SSVEP+EOG case. The class numbers (output class and target class) in the horizontal and vertical axes indicate the position illustrated at the top panel of the figures. C1, C2, and C3 represent the frequency configurations depicted in Figure 3C.

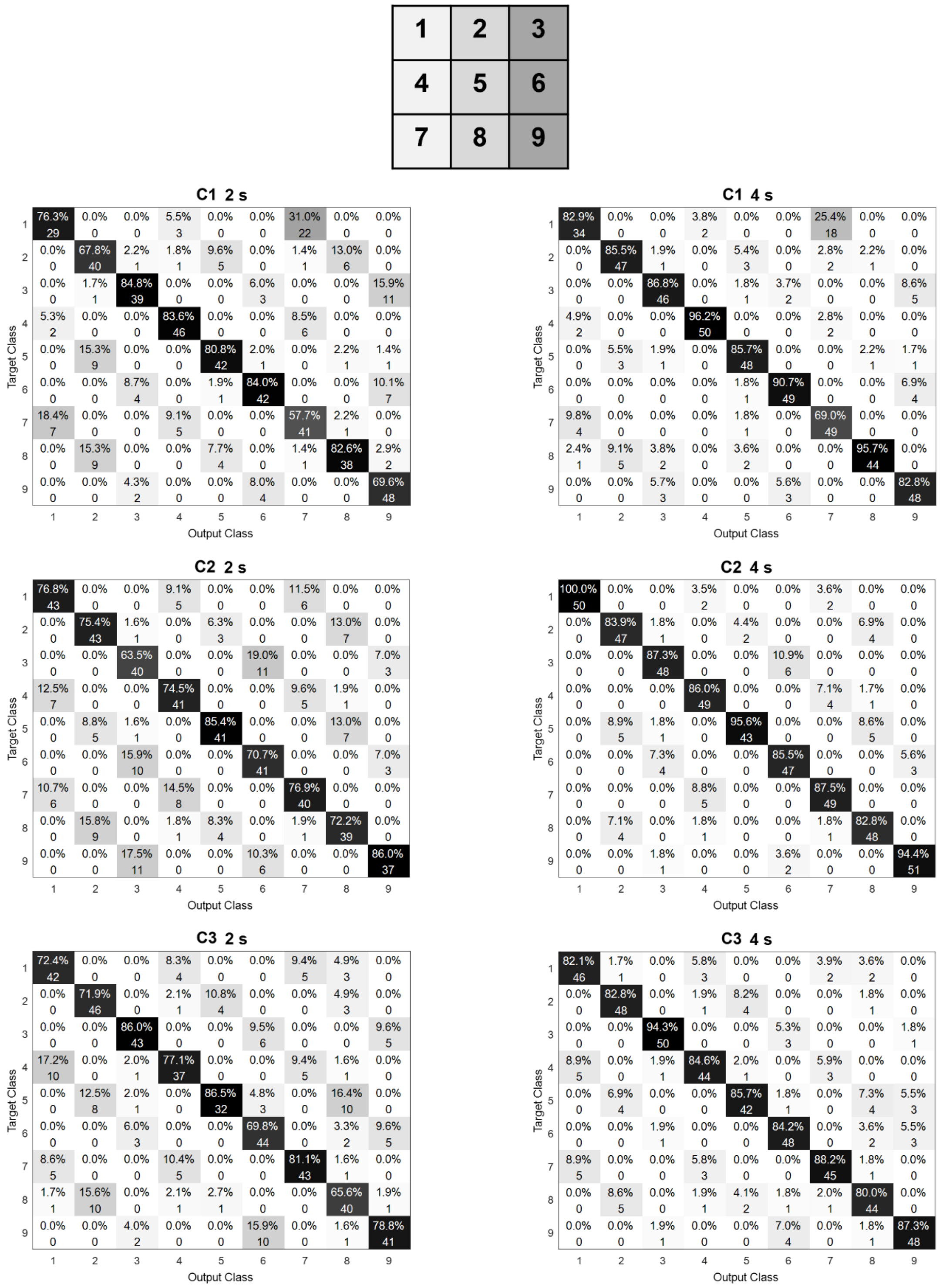

Supplement: Supplementary file 1 [file Data_Sheet_1.PDF]
